# A retrospective survey of the seroprevalence of severe fever with thrombocytopenia syndrome virus in wild animals in Japan

**DOI:** 10.1002/vms3.400

**Published:** 2020-11-29

**Authors:** Ayaka Okada, Akitoyo Hotta, Masanobu Kimura, Eun‐sil Park, Shigeru Morikawa, Yasuo Inoshima

**Affiliations:** ^1^ Laboratory of Food and Environmental Hygiene Cooperative Department of Veterinary Medicine Faculty of Applied Biological Sciences Gifu University Gifu Japan; ^2^ Education and Research Center for Food Animal Health Gifu University (GeFAH) Gifu Japan; ^3^ Department of Veterinary Science National Institute of Infectious Diseases Shinjuku Tokyo Japan; ^4^ The United Graduate School of Veterinary Sciences Gifu University Gifu Japan; ^5^ Joint Graduate School of Veterinary Sciences Gifu University Gifu Japan

**Keywords:** seroprevalence, severe fever with thrombocytopenia syndrome, wild animal

## Abstract

Severe fever with thrombocytopenia syndrome (SFTS) has a high fatality rate and is caused by SFTS virus (SFTSV). Currently, SFTS is endemic to some areas in western Japan, and wild animals are considered to play important roles in the circulation of SFTSV in the environment. Previous retrospective surveys using samples mainly obtained between 2006 and 2015 revealed serological evidence of SFTSV infection in wild animals; however, seroprevalence before 2006 remains unclear. In this study, we investigated the presence of anti‐SFTSV antibodies in a total of 521 serum samples from nine wild animal species collected from 11 prefectures in central and eastern Japan between 1980 and 2000. All samples yielded negative results for antibodies to SFTSV, suggesting that there had been few or no SFTSV infections before 2000 in the sampled areas.

## Graphical abstract

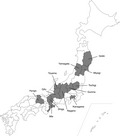

## 1 INTRODUCTION

Severe fever with thrombocytopenia syndrome (SFTS) is an emerging viral disease with a high fatality rate and is caused by SFTS virus (SFTSV), which was recently renamed Dabie bandavirus by the International Committee on Taxonomy of Viruses (Liang et al., 2019). SFTSV belongs to the genus *Bandavirus* in the family *Phenuiviridae*, order *Bunyavirales* (Liang et al., 2019). SFTS was first reported in China in 2011 (Yu et al., 2011), and subsequently, in South Korea and Japan (Kim et al., 2013; Takahashi et al., 2014). SFTSV isolated in Japan and China formed different clusters in a phylogenetic tree (Takahashi et al., 2014). In addition, several human serum samples obtained between 2005 and 2012 in Japan tested positive for SFTSV (Takahashi et al., 2014), suggesting that SFTSV might have existed in Japan before its appearance of SFTS in China. Humans are infected with SFTSV by the bite of an infected tick (Zhang et al., 2012). In addition, human‐to‐human transmission can occur via direct contact with blood or bloody secretions from infected humans (Gai et al., 2012). It was reported that cat‐to‐human transmission occurs via direct contact with SFTSV‐infected cats (Kida et al., 2019). Thus, infected mammals, possibly including wild animals, could be a source of infection for humans. In Japan, there have been several reports on SFTSV‐seropositive wild animals (Fig. S1). In Nagasaki Prefecture, where the first SFTSV‐seropositive human was reported, SFTSV antibodies were also found in 36 of 190 (18.9%) Japanese wild boars (*Sus scrofa leucomystax*) between 2006 and 2012 (Hayasaka et al., 2016). SFTSV antibodies were also detected in 5 of 20 (25%) Japanese deer (*Cervus nippon centralis*) and in 10 of 40 (25%) Japanese wild boars in Ehime Prefecture in 2013 and 2014 (Kimura et al., 2018). Previous retrospective surveys have shown serological evidence of SFTSV infection in wild animals since 2006, including Japanese badger (*Meles meles anakuma*), Japanese deer, Japanese macaque (*Macaca fuscata*), Japanese raccoon dog (*Nyctereutes procyonoides viverrinus*), Japanese wild boar and masked palm civet (*Paguma larvata*) (Hayasaka et al., 2016; Kimura et al., 2018; Lundu et al., 2018; Morikawa et al., 2016; Uchida et al., 2018). In these studies, all of the seropositive animals were reported in western Japan, an SFTS‐endemic region for humans. Therefore, wild animals are considered to play important roles in the maintenance and spread of SFTSV in the environment. However, it is still unclear whether SFTSV was present in Japan before 2005, the year in which SFTSV was first detected in human serum samples (Takahashi et al., 2014). It is important to demonstrate when SFTSV first appeared in Japan, and whether SFTSV‐seropositive wild animals exist in the areas other than western Japan. The Ministry of the Environment indicated that the numbers of hunted animals gradually increased recently, and 561,000 Japanese deer and 602,200 wild boars were hunted in Japan in the fiscal year 2018 (Ministry of the Environment, 2019). Therefore, it is necessary to verify the risk of SFTSV infection in humans involved in the handling of hunted wild animals. In the present study, we investigated the presence of antibodies to SFTSV in serum samples from wild animals collected in central and eastern Japan between 1980 and 2000.

## 2 MATERIALS AND METHODS

**FIGURE 1:**
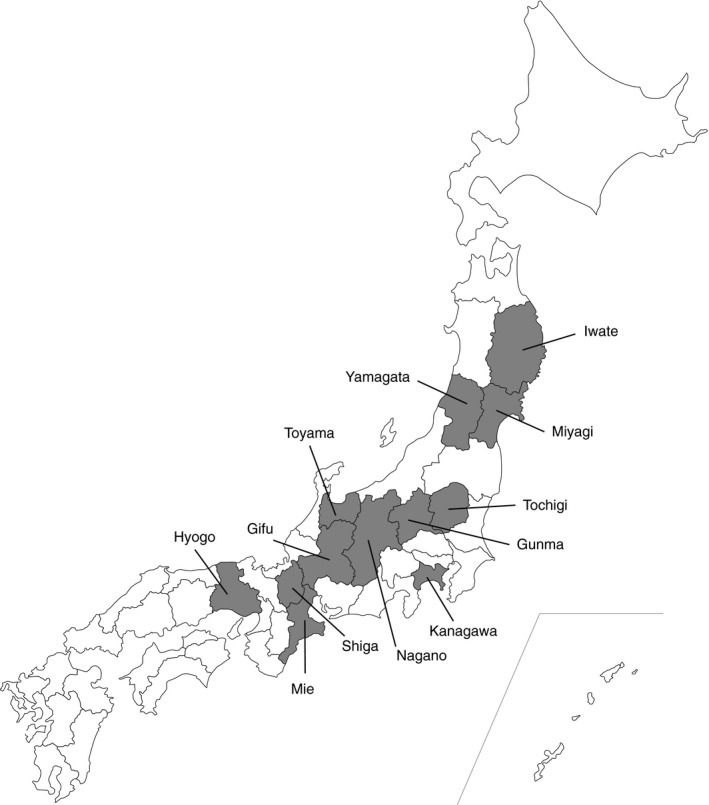
Map of Japan showing prefectures (grey) where a total of 521 serum samples from nine wild animal species were collected. The date of collection is as follows. Gifu; Japanese badger (1992), Japanese black bear (1991–1993), Japanese macaque (1991, 1992), Japanese racoon dog (1991, 1992), Japanese serow (1980–1985, 1999), Japanese wild boar (1991, 1992), Masked palm civet (1992), Nutria (1991, 1992), Shiga; Japanese black bear (1991, 1992), Japanese wild boar (1991, 1992), Hyogo; Japanese deer (1992), Japanese wild boar (1992), Iwate; Japanese deer (1992), Gunma; Japanese deer (1999), Mie; Japanese racoon dog (1992), Kanagawa; Japanese serow (1989), Tochigi; Japanese serow (1991–1993), Yamagata; Japanese serow (1991–1999), Toyama; Japanese serow (1999), Nagano; Japanese serow (1999)

A total of 521 serum samples were collected from nine wild animal species in 11 prefectures throughout the largest island of Japan, Honshu, which were used in previous studies ( Inoshima et al., 1999, 2001, 2002; Suzuki et al., 1993) (Table 1 and Table S1, and Figure 1). The nine species were Japanese badger, Japanese black bear (*Ursus thibetanus japonicus*), Japanese deer, Japanese macaque, Japanese raccoon dog, Japanese serow (*Capricornis crispus*), Japanese wild boar, masked palm civet and nutria (*Myocastor coypus*). These samples were heat‐inactivated at 56°C for 30 min.

**TABLE 1:** Wild animals tested for antibodies to severe fever with thrombocytopenia syndrome virus

Animals	Genus and species	No. of positive/ No. of serum samples tested (Prefecture) [Reference]		
		This study		Previous studies^a^
		1980–1989	1990–2000	2006–2016
Japanese badger	*Meles meles anakuma*		0/2 (Gifu)	6/74 (Wakayama) ( Morikawa et al., 2016; Shimoda & Maeda, 2015)
Japanese black bear	*Ursus thibetanus japonicus*		0/26 (Gifu)	
			0/3(Shiga)	
Japanese deer	*Cervus nippon centralis*		0/3 (Iwate)	5/20 (Ehime) ( Kimura et al., 2018)
			0/27 (Hyogo)	+ (Hyogo) ( Morikawa et al., 2014)
			0/50 (Gunma)	+ (Gunma, Miyagi, Nagano, Shizuoka, Tochigi, Yamanashi) ( Lundu et al., 2018; Morikawa et al., 2016)
				1/9 (Wakayama) ( Morikawa et al., 2016; Shimoda & Maeda, 2015)
				217/502 (Yamaguchi) ( Morikawa et al., 2016)
Japanese macaque	*Macaca fuscata*		0/30 (Gifu)	3/15 (Wakayama) ( Morikawa et al., 2016; Shimoda & Maeda, 2015)
Japanese raccoon dog	*Nyctereutes procyonoides viverrinus*		0/23 (Gifu)	39/531 (Wakayama) ( Morikawa et al., 2016; Shimoda & Maeda, 2015)
			0/1 (Mie)	
Japanese serow	*Capricornis crispus*	0/1 (Kanagawa)	0/4(Gifu)	
		0/150 (Gifu)	0/1 (Nagano)	
			0/10 (Tochigi)	
			0/1 (Toyama)	
			0/124 (Yamagata)	
Japanese wild boar	*Sus scrofa leucomystax*		0/8 (Gifu)	10/40 (Ehime) ( Kimura et al., 2018)
			0/1 (Hyogo)	36/190 (Nagasaki) ( Hayasaka et al., 2016)
			0/2 (Mie)	2/89 (Wakayama) ( Morikawa et al., 2016; Shimoda & Maeda, 2015)
			0/19 (Shiga)	32/370 (Yamaguchi) ( Morikawa et al., 2016)
Masked palm civet	*Paguma larvata*		0/5 (Gifu)	5/16 (Wakayama) ( Morikawa et al., 2016; Shimoda & Maeda, 2015)
Nutria	*Myocastor coypus*		0/30 (Gifu)	

^a^ +, seropositive animals, but not exact numbers, were reported.

Titres of antibodies to SFTSV antigen were determined using an enzyme‐linked immunosorbent assay (ELISA) using protein A/G conjugated with horseradish peroxidase. Briefly, half of the wells of the ELISA plates (Nunc, Roskilde, Denmark) were coated with a predetermined optimal quantity (approximately 100 ng/well) of lysates of SFTSV‐infected Huh7 cells, and the remaining wells were coated with negative control antigens (lysates of Huh7 mock‐infected cells). The antigens used in the ELISAs were prepared as described previously (Kimura et al., 2018; Park et al., 2019). The antigen‐coated plates were incubated at 4°C overnight, washed with phosphate‐buffered saline (PBS) containing 0.05% Tween‐20 (PBS‐T), incubated with 200 μl/well of PBS‐T containing 20% Blocking One (Nacalai Tesque, Kyoto, Japan) for 1 hr at 37°C. The wells were washed three times with PBS‐T, inoculated with the test samples (100 μl/well) in PBS‐T containing 10% Blocking One at dilutions of 1 in 100, 400, 1,600 and 6,400 in a single experiment, incubated for 1 hr at 37°C, washed and incubated with a Pierce Recombinant Protein A/G, Peroxidase Conjugated (Life Technologies, Rockford, IL, USA) at a dilution of 1 in 10,000 in PBS‐T for 1 hr at 37°C. Subsequently, the plates were washed three times, and 100 μl/well of 2,2'‐azinobis (3‐ethylbenzothiazoline‐6‐sulfonic acid) (ABTS) solution (Roche Diagnostics, Mannheim, Germany) was added, and the plates were incubated for 30 min at room temperature. Absorbance was measured using an iMark microplate reader (Bio‐Rad, Hercules, CA, USA) at 405 nm. Optical densities (ODs) were calculated by subtracting the ODs of the negative control antigen‐coated wells from those of the corresponding SFTSV‐infected cell antigen‐coated wells. A serum sample collected from Japanese deer in Yamaguchi Prefecture in 2013 (Morikawa et al., 2016) was used as a reference control for each test. OD values in each experiment were adjusted by calculating the OD value of the reference serum to SFTSV‐infected Huh7 cells at a dilution of 400 as 2.0. By this adjustment, the expected OD values for all samples were determined. A sample was considered ELISA‐positive if the adjusted OD value exceeded the cut‐off value (mean + 3SD). Negative control sera of normal rabbits, rats and mice typically showed OD values between −0.14 and 0.13 in this assay.

## 3 RESULTS AND DISCUSSION

In every experiment, reference control sera showed high OD values for SFTSV‐antigen at a dilution of 1 in 100 and 400. In every experiment, the signal/background ratios were above 5.5. After subtraction of the standardized background OD values, the reference sample remained at OD values between 1.37 and 1.85 in all experiments. However, all of the 521 samples (Table 1), showed low reactivity, and their background subtraction was below the cut‐off value (0.33). The presence of antibodies to SFTSV in wild animals was considered as evidence of past SFTSV infection. Thus, these results indicated that SFTSV infection was non‐existent or rare before 2000, in these animal species in the tested areas in Japan.

In the present study, SFTSV antibodies were not detected in 27 Japanese deer in Hyogo Prefecture. In 2013, SFTSV antibodies were detected in Japanese deer in Hyogo Prefecture (Morikawa et al., 2014) (Table 1), suggesting that SFTSV infection of the Japanese deer population in Hyogo Prefecture occurred between 1992 and 2013. Although the exact years and the numbers of infected deer were not indicated, it was reported that SFTSV antibodies were detected in Japanese deer in several prefectures in central and eastern Japan (Miyagi, Tochigi, Gunma, Nagano, Yamanashi, and Shizuoka Prefectures) between 2007 and 2015 (Morikawa et al., 2016) (Table 1). Our study indicated that there were no SFTSV antibodies in Gunma Prefecture in 1999. Investigating the seroprevalence of SFTSV in these prefectures using old serum samples obtained in the period between our study and the previous study would be required to clarify the timeline of SFTSV infection of the Japanese deer population in central and eastern Japan.

SFTSV antibodies were also detected in Japanese wild boars in Nagasaki Prefecture, whose blood samples were collected between 2006 and 2012 (Hayasaka et al., 2016), and in Wakayama Prefecture between 2011 and 2015 (Morikawa et al., 2016; Shimoda & Maeda, 2015) (Table 1). However, SFTSV antibodies were not detected in Japanese wild boars in Wakayama Prefecture in 2010 ( Morikawa et al., 2016; Shimoda & Maeda, 2015). It is conceivable that SFTSV infection of the Japanese wild boar population in Nagasaki Prefecture occurred during 1992–2012, and then spread to the population in Wakayama Prefecture in 2010. In the present study, SFTSV antibodies were not detected in 30 Japanese wild boars in Gifu, Hyogo, Mie and Shiga Prefectures between 1990 and 2000. Because these prefectures are close to Wakayama Prefecture, serum samples obtained sometime around 2010 would be required to clarify when SFTSV infected the Japanese wild boar population in these prefectures.

In Wakayama Prefecture, serum samples of Japanese raccoon dogs collected between 2007 and 2015 were SFTSV seronegative until 2009 but were found to be seropositive since 2010 (Morikawa et al., 2016; Shimoda & Maeda, 2015) (Table 1), suggesting that SFTSV might have infected the Japanese raccoon dog population between 2009 and 2010 in Wakayama Prefecture. However, our data indicated that no SFTSV antibodies were detected in any of the 24 Japanese raccoon dog samples obtained from Gifu and Mie Prefectures in 1992. Because Gifu and Mie Prefectures are close to Wakayama Prefecture, it is not possible to confirm which prefecture was the first among these to have wild animals infected with SFTSV. Serum samples obtained sometime around 2010 would be required to clarify the timeline of SFTSV infection of the raccoon dog population in these prefectures.

SFTSV antibodies were also detected in Japanese badgers, Japanese macaque, and masked palm civets in Wakayama Prefecture between 2007 and 2015 (Morikawa et al., 2016; Shimoda & Maeda, 2015) (Table 1). SFTSV might have infected Japanese badger, Japanese macaque and masked palm civet population between 1992 and 2007. Thus, SFTSV antibodies have not been reported in Japanese black bear, Japanese serow and nutria. This might be because these animal species have little or no susceptibility to SFTSV, or are highly susceptible to SFTSV, leading to the rapid death of the infected animals. Further studies on these three animal species are needed to determine their degree of susceptibility to SFTSV and to monitor the transmission of SFTSV among them.

In conclusion, in the present retrospective study, we did not find SFTSV infections in any of the nine wild animal species examined between 1980 and 2000 in central and eastern Japan. Thus, the humans who handled the wild animals in previous studies (Inoshima et al., 1999, 2001, 2002; Suzuki et al., 1993) might have had a low risk of acquiring SFTSV infection from these wild animals. As described above, the previous studies indicated that SFTSV antibodies were first detected in 2010 in western Japan, and between 2007 and 2015 in eastern Japan. Further studies using old serum samples collected from wild animals are needed to determine the exact timeline of SFTSV infection of wild animals in Japan. Because our samples were collected from a limited number of regions, and the individual sample sizes are small, further studies with extensive sample collection from different regions are required. Investigating the existing samples that were used in other studies, which are similar to the present study, is important to verify the timeline of the SFTSV infection of wild animals in Japan.

## CONFLICTS OF INTEREST

The authors declare that there are no conflicts of interest.

## AUTHOR CONTRIBUTION

Ayaka Okada: Data curation; Investigation; Writing‐original draft. Akitoyo Hotta: Investigation; Methodology; Writing‐original draft. Masanobu Kimura: Methodology. Eunsil Park: Methodology. Shigeru Morikawa: Funding acquisition; Writing‐review & editing. Yasuo Inoshima: Conceptualization; Funding acquisition; Project administration; Resources; Supervision; Writing‐review & editing.

## ETHICAL STATEMENT

Sample collection was carried out according to the animal welfare code of Japan.

### PEER REVIEW

The peer review history for this article is available at https://publons.com/publon/10.1002/vms3.400.

## Supporting information

Figure S1

Table S1
