# Supplementary figures and images for: A retrospective survey of the seroprevalence of severe fever with thrombocytopenia syndrome virus in wild animals in Japan
Source: Vet Med Sci. 2020 Nov 29;7(2):600–5. doi: 10.1002/vms3.400 (PMC8025650; doi:10.1002/vms3.400)

Supplementary Fig. S1.

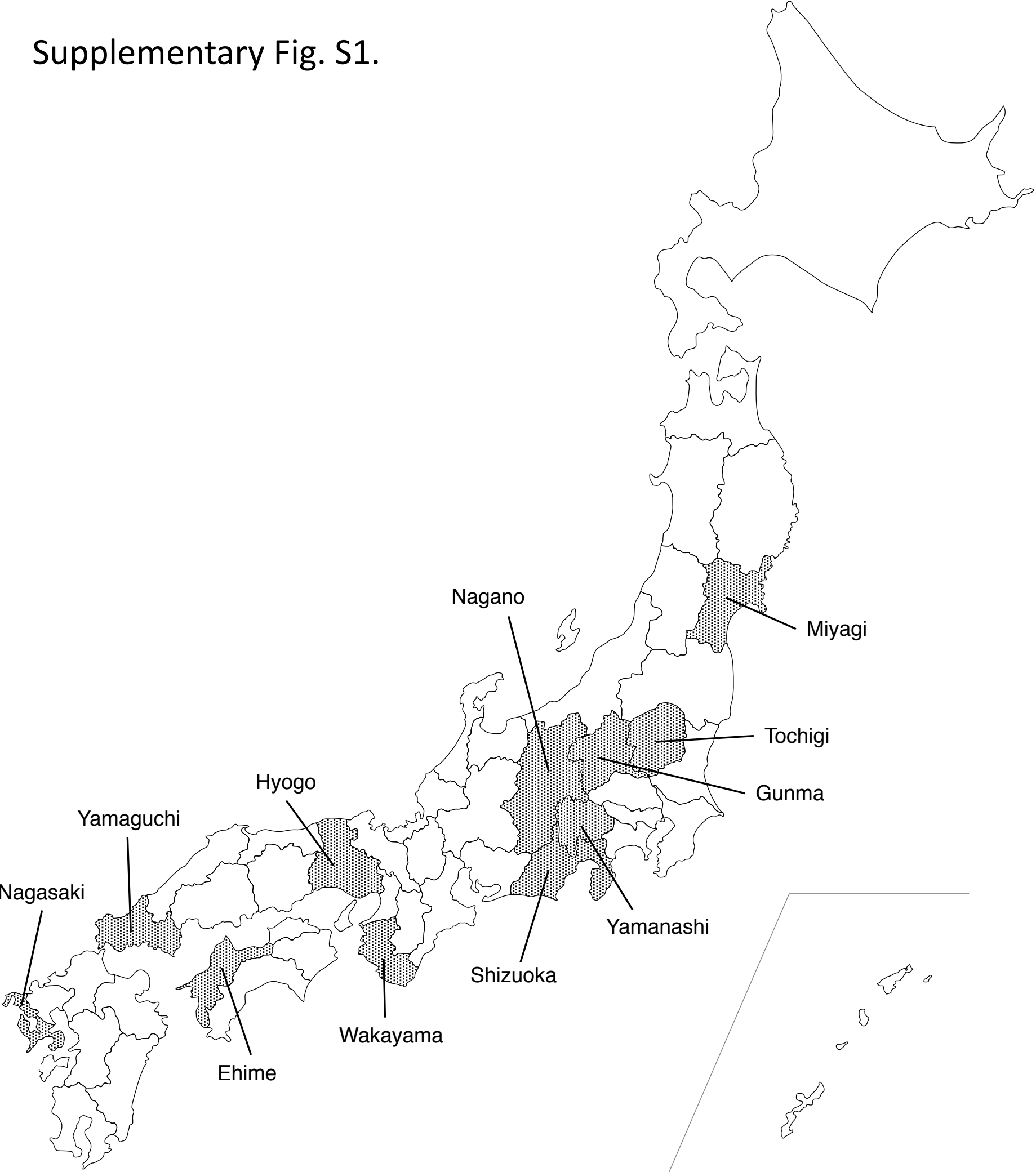

Supplement: Supplementary file 1 — Figure S1 [file VMS3-7-600-s002.pdf]
