# Supplementary material for: A retrospective survey of the seroprevalence of severe fever with thrombocytopenia syndrome virus in wild animals in Japan
Source: Vet Med Sci. 2020 Nov 29;7(2):600–5. doi: 10.1002/vms3.400 (PMC8025650; doi:10.1002/vms3.400)
Supplement: Supplementary file 2 — Table S1 [file VMS3-7-600-s001.pdf]

Supplement Table S1. Information of the wild animals tested for antibodies to severe fever with thrombocytopenia syndrome virus

Animal	Genus and species	No. of serum samples tested	Period of serum collection	Prefectures where sera were collected
Japanese badger	*Meles meles anakuma*	2	1992	Gifu
Japanese black bear	*Ursus thibetanus japonicus*	2	1991	Shiga
		2	1991	Gifu
		1	1992	Shiga
		17	1992	Gifu
		7	1993	Gifu
Japanese deer	*Cervus nippon centralis*	27	1992	Hyogo
		3	1992	Iwate
		50	1999	Gunma
Japanese macaque	*Macaca fuscata*	24	1991	Gifu
		6	1992	Gifu
Japanese raccoon dog	*Nyctereutes procyonoides viverrinus*	4	1991	Gifu
		1	1992	Mie
		19	1992	Gifu
Japanese serow	*Capricornis crispus*	30	1980	Gifu
		18	1981	Gifu
		12	1982	Gifu
		18	1982	Gifu
		24	1983	Gifu
		18	1984	Gifu
		11	1984	Gifu
		19	1985	Gifu
		1	1989	Kanagawa
		2	1991	Tochigi
		3	1991	Yamagata
		6	1992	Yamagata
		5	1992	Tochigi
		5	1993	Yamagata
		3	1993	Tochigi
		5	1994	Yamagata
		6	1995	Yamagata
		23	1996	Yamagata
		14	1997	Yamagata
		32	1998	Yamagata
		3	1999	Gifu
		1	1999	Toyama
		1	1999	Nagano
		1	1999	Yamagata
		29	1999	Yamagata
		1	2000	Gifu
Japanese wild boar	*Sus scrofa leucomystax*	3	1991	Gifu
		6	1991	Shiga
		5	1992	Gifu
		2	1992	Mie
		13	1992	Shiga
		1	1992	Hyogo
Masked palm civet	*Paguma larvata*	5	1992	Gifu
Nutria	*Myocastor coypus*	8	1991	Gifu
		22	1992	Gifu
